# Comparative analysis of the prevalence of the glutathione S-transferase (GST) system in malignant and benign thyroid tumor cells

**DOI:** 10.1590/S1516-31802007000500008

**Published:** 2007-09-02

**Authors:** Lucia Helena de Carvalho, Kauê Serdeira, Marianne Yumi Nakai, Tatiana Ramos Malavasi

**Keywords:** Thyroid gland, Neoplasms, Glutathione S-transferase pi, Molecular biology, Prognosis, Glândula tireóide, Neoplasia, Glutationa S-transferase pi, Biologia molecular, Prognóstico

## Abstract

**CONTEXT AND OBJECTIVE:**

When null, the *mu* and *theta* genes of the glutathione S-transferase system (GSTM1 and GSTT1, respectively) are related to malignant tumors affecting the lungs, colon, prostate, bladder and head and neck. In the thyroid, the appearance of cancer has been correlated with deletion of these genes. The aim of this study was to compare the frequencies of these genes in patients with benign and malignant tumors of the thyroid gland.

**DESIGN AND SETTINGS:**

This was a cross-sectional clinical trial carried out in the Head and Neck Surgery Division, Faculdade de Medicina da Santa Casa de São Paulo.

**METHODS:**

Samples of thyroid tissue were collected from 32 patients and divided into two groups: benign tumor (A) and malignant tumor (B). After DNA extraction, the genes were amplified using PCR.

**RESULTS:**

The B group presented four cases of positive genotyping for both genes, seven positive for GSTT1 and negative for GSTM1, two negative for GSTT1 and positive for GSTM1, and only one case of double negative. The A group showed 11 cases with positive genotyping for both genes and none with the double negative genotype.

**CONCLUSION:**

In this study, there was no relationship between the presence of the GSTT1 and GSTM1 genes and the benign and malignant thyroid tumors.

## RESUMO

**CONTEXTO E OBJETIVO:**

Os genes do sistema glutationa S-transferase *mu* e *theta* (GSTM1 e GSTT1, respectivamente), quando nulos, apresentam relação com tumores malignos de pulmão, cólon, próstata, bexiga e cabeça e pescoço, podendo nesses casos ser utilizados como marcadores tumorais. Na tireóide, o surgimento do câncer tem sido relacionado à deleção desses genes. Assim, o objetivo deste estudo foi comparar a freqüência dos genes GSTM1 e GSTT1 em pacientes com tumores benignos e malignos da glândula tireóide.

**TIPO DE ESTUDO E LOCAL:**

Estudo clínico transversal, realizado na Faculdade de Ciências Médicas da Santa Casa de São Paulo.

**MÉTODOS:**

Amostras de tecido tireoidiano foram coletados de 32 pacientes e divididas em dois: tumor benigno (A) e carcinoma (B). Após extração do DNA os genes foram amplificados em reação de polimerase em cadeia.

**RESULTADOS:**

O grupo B apresentou 4 casos de genótipo positivo para ambos os genes, 7 positivos para GSTT1 e negativos para GSTM1, 2 negativos para GSTT1 e positivos para GSTM1, e apenas 1 caso duplo negativo. Já o grupo A mostrou 11 casos com genótipo positivo para ambos os genes e nenhum com o genótipo duplo negativo.

**CONCLUSÃO:**

Não há relação entre a presença dos genes GSTT1 e GSTM1 com o carcinoma bem diferenciado e os tumores benignos da tireóide em nossos casos.

## INTRODUCTION

Human neoplasia occurs by means of a genetic non-lethal lesion in a single stem cell. By multiplying, this damaged cell gives rise to the tumor. These cellular abnormalities, which are advantageous to the cell itself and disadvantageous to the organism, occur because of acquired or inherited genetic alterations that alter the expression of the biochemical properties of the genes, particularly of those involved in the cycle or maintenance of cellular life.^1^

The appearance of malignant lesions in the thyroid is related to the deletion of the glutathione S-transferase (GST) *theta* and *mu* genes (GSTT1 and GSTM1, respectively).^2^ GST consists of a large multigenic group of detoxification enzymes.^3^ GSTs catalyze the conjugation of toxic compounds with glutathione (GSH -γ-glutamyl cysteinyl glycine, which is a tripeptide), thereby inactivating hazardous effects in the cell. These protein dimers conjugate with electrolytic molecules and free radicals in order to reduce them to products that are nontoxic to the organism. The deletion of GSTT1 and GSTM1 increases the risk of thyroid lesions by a factor of 2.6.^4^

## OBJECTIVE

The aim of the present study was to compare GSTM1 and GSTT1 gene frequencies between patients with benign and malignant tumors of the thyroid gland.

## METHODS

### Setting

This was a cross-sectional study that analyzed 32 patients who underwent operations performed by the Head and Neck Surgery Group at Hospital da Irmandade de Misericórdia da Santa Casa de São Paulo between October 2005 and September 2006 after approval by the institution's Ethics Committee.

### Sample

Thyroid tissue samples were collected consecutively from 32 patients and evaluated with regard to the presence or absence of the GSTT1 and GSTM1 genes. There were 29 female patients (90.6%) and three male patients (9.4%). The patients were divided into two groups, benign tumors (A) and malignant tumors (B), based on the anatomopathological study. There were 18 female patients in the A group. In the B group, there were 3 males (21.4%) and 11 females (78.6%); of these 14 patients, two had follicular carcinoma and 12 had papillary carcinoma (Table 1).

**Table 1.** Types of thyroid neoplasia in relation to patients’ gender

Gender	Benign tumor	Carcinoma		Total
		Papillary	Follicular	
Male	0	2	1	**3**
Female	18	10	1	**29**
**Total**	**18**	**12**	**2**	**32**

### Procedures

Immediately after thyroid ablation, before the surgery procedures finished, the nodules were divided into two halves: one fragment was stored in liquid nitrogen (N_2_) and then in a freezer at −80° C, and the other was sent for anatomopathological study, in 10% formol. To extract the thyroid DNA, the Qiagen DNeasy^®^ kit was used. The GSTM1 and GSTT1 genes were amplified using the polymerase chain reaction (PCR), including amplification of the β-globin gene as a positive control for the DNA sample, and then the genotype was identified by viewing under ultraviolet light.

### Statistical analysis

The statistical method used was Fisher's exact test, with a confidence interval of 95% (p < 0.05).

## RESULTS

After DNA extraction, we observed the PCR pattern that is shown in Table 2. In group A, there were no cases of double negati-ve genotype, while 61.1% were double positive and 38.9% were positive for one of the genes. In group B, one of the cases presented double negative genotype, while the others were distributed between double positive (28.6%), positive genotyping only for GSTT1 (50%) and positive only for GSTM1 (14.3%).

**Table 2.** Results from DNA analysis of thyroid tumors patients

	Diagnoses		
Genotype	Adenoma	Carcinoma	Total
T + M +	11	4	**15**
T + M -	5	7	**12**
T - M +	2	2	**4**
T - M -	0	1	**1**
**Total**	**18**	**14**	**32**

*T = genotype of GSTT! gene; M = genotype of GSTM! gene; + = present; - = absent.*

## DISCUSSION

The data collected for analyzing the benign tumor group showed high frequency of the double positive genotype (61.1%) and no double negative genotype. In group B there was a more homogeneous distribution. Cases with positive GSTT1 genotype and negative GSTM1 (T + M -; 50%) predominated, while the double negative genotype was observed in only one case (7.1%). The double positive genotype occurred in 28.6%.

Analysis of the GSTM1 gene alone showed that there was a relatively high frequency of the negative GSTM1 genotype in group B, i.e. 8 out of the 14 cases (57.1%), in comparison with group A, in which the frequency was 27.8%. However, statistical analysis showed that this was not significant (p = 0.09). Analysis of the GSTT1 gene alone showed that it was present in 88.9% of the cases in group A and 78.6% of the cases in group B, and also was not statistically significant (p = 0.3).

In the literature, separate analyses of the genes GSTT1 and GSTM1 are inconclusive regarding the risk of developing thyroid cancer.^5^ Our results from separate analyses of these genes are in accordance with the literature, although we observed the presence of GSTM1 in 72.2% (13/18) and GSTT1 in 88.9% (16/18) of the benign tumor cases. These frequencies are quite high and, although non-significant, cannot be ignored.

## CONCLUSION

Analysis of the data obtained, under the conditions of the present study, allows the conclusion that there is no relationship between the presence of the GSTT1 and GSTM1 genes and the benign and malignant thyroid tumors.

However, the relatively small number of cases makes it impossible to reach more substantial conclusions. In order to achieve this, a larger sample would be needed.

## References

[1] Namba H, Rubin SA, Fagin JA (1990). Point mutations of ras oncogenes are an early event in thyroid tumorigenesis. Mol Endocrinol.

[2] Morari EC, Leite JL, Granja F, da Assumpção LV, Ward LS (2002). The null genotype of glutathione s-transferase M1 and T1 locus increases the risk for thyroid cancer. Cancer Epidemiol Biomarkers Prev.

[3] Widersten M, Mannervik B (1995). Glutathione transferases with novel active sites isolated by phage display from a library of random mutants. J Mol Biol.

[4] Haq M, Harmer C (2004). Thyroid cancer: an overview. Nucl Med Commun.

[5] Hernández A, Céspedes W, Xamena N (2003). Glutathione S-transferase polymorphisms in thyroid cancer patients. Cancer Lett.

